# A competing-risk-based score for predicting twenty-year risk of incident diabetes: the Beijing Longitudinal Study of Ageing study

**DOI:** 10.1038/srep37248

**Published:** 2016-11-16

**Authors:** Xiangtong Liu, Zhenghong Chen, Jason Peter Fine, Long Liu, Anxin Wang, Jin Guo, Lixin Tao, Gehendra Mahara, Kun Yang, Jie Zhang, Sijia Tian, Haibin Li, Kuo Liu, Yanxia Luo, Feng Zhang, Zhe Tang, Xiuhua Guo

**Affiliations:** 1 School of Public Health, Capital Medical University, Beijing 100069, China; 2 Beijing Municipal Key Laboratory of Clinical Epidemiology, Beijing 100069, China; 3 Beijing Neurosurgical Institute, Capital Medical University, 6, Tiantanxili, Beijing, 100050, China; 4 Department of Biostatistics, University of North Carolina, Chapel Hill, 46200, NC, U.S.A; 5 Department of Statistics & Operations Research, University of North Carolina, Chapel Hill, 319200, NC, U.S.A; 6 Beijing Geriatric Clinical and Research Center, Xuanwu Hospital, Capital Medical University, Beijing 100053, China

## Abstract

Few risk tools have been proposed to quantify the long-term risk of diabetes among middle-aged and elderly individuals in China. The present study aimed to develop a risk tool to estimate the 20-year risk of developing diabetes while incorporating competing risks. A three-stage stratification random-clustering sampling procedure was conducted to ensure the representativeness of the Beijing elderly. We prospectively followed 1857 community residents aged 55 years and above who were free of diabetes at baseline examination. Sub-distribution hazards models were used to adjust for the competing risks of non-diabetes death. The cumulative incidence function of twenty-year diabetes event rates was 11.60% after adjusting for the competing risks of non-diabetes death. Age, body mass index, fasting plasma glucose, health status, and physical activity were selected to form the score. The area under the ROC curve (AUC) was 0.76 (95% Confidence Interval: 0.72–0.80), and the optimism-corrected AUC was 0.78 (95% Confidence Interval: 0.69–0.87) after internal validation by bootstrapping. The calibration plot showed that the actual diabetes risk was similar to the predicted risk. The cut-off value of the risk score was 19 points, marking mark the difference between low-risk and high-risk patients, which exhibited a sensitivity of 0.74 and specificity of 0.65.

Diabetes is a well-recognized cause of premature death and disability and is associated with an increased risk of kidney failure, cardiovascular disease, lower-limb amputation and blindness^1^. Diabetes was directly responsible for 1.5 million deaths and 89 million disability-adjusted life years in 2012. The prevalence of diabetes is increasing in all populations worldwide, and at a particularly accelerated rate in low- and middle-income countries^2^. China has had the largest absolute disease burden because of its large population base^3^. The financial costs of diabetes-related health expenditures also pose a substantial burden on the nation’s economy. Older adults are one of the fastest growing age groups both worldwide and in Beijing city^4^. By the end of 2008, the total population of Beijing had reached 12.3 million, with the number of people aged over 60, over 65, and over 80 years being 2.18, 1.62, and 0.29 million, representing 17.7%, 13.2%, and 2.4% of the total population, respectively^5^.

However, the test generally used to identify high-risk subjects, the 2-hour oral glucose tolerance test, is limited by its invasive and time-consuming nature and its relatively high costs^6^. Blood glucose has a large random variation and only provides information on a subject’s current glycemic state. In the past few years, researchers have been able to construct multivariable risk tools that are intended to aid clinicians in conducting risk assessments. The application of risk assessment tools to screen high-risk subjects based on demographic and anthropometric characteristics and simple laboratory tests is both feasible and economical^7^.

There are a number of risk assessment tools based on readily available clinical variables that predict the development of new diabetes cases, including ones proposed by the Framingham Offspring study^8^, Rancho Bemardo study^9^, and Guangzhou Biobank Cohort study^10^. The available tools have been derived from European^7^,^8^,^11^,^12^,^13^, American^6^,^8^,^9^,^14^, Australian^15^, Brazilian^16^, Africa^17^, and Asian^10^,^18^,^19^,^20^,^21^ populations. Differences in ethnicities, locations, and lifestyles partly limit the applicability of some of the effective risk scores to the Chinese population. Despite the large number of risk tools being developed, only a very small minority are designed for middle-aged and older Asian populations, Chinese populations in particular^8^,^10^. In addition, few diabetes risk prediction models included health status, despite the fact that some studies have confirmed that health status is an important diabetes predictor^22^. Furthermore, current diabetes risk prediction algorithms were developed for 10-year periods or less. The increasing life expectancy and elderly population suggest the need for longer-term risk assessment tools.

Additionally, conventional statistical methods that analyse time-to-event data assume an absence of competing risks^23^. However, competing risks must be explicitly considered explicitly in frail populations, especially among the elderly^24^. Ignoring the presence of competing risks can bias estimates of the incidence of the event of interest upwards^24^,^25^,^26^. Specifically, the sum of the estimates of each event type’s incidence will exceed the estimates of the incidence of the composite outcome, defined as any of the event types^25^. To overcome this problem, sub-distribution hazards models (e.g., Fine-Gray model) were proposed, in which the cumulative incidence function (CIF) is provided to estimate the incidence of an event while accounting for the presence of competing events^25^. Sub-distribution hazards models permit one to assess the association of predictors on the absolute risk of DM as well as to calculate the absolute risk of DM conditionally on those predictors. Sub-distribution hazards models are being increasingly applied to predict diseases^26^,^27^. However, to the best of our knowledge, no algorithm has been proposed that quantifies the 20-year risk of diabetes among middle-aged and elderly individuals using a sub-distribution hazards model.

In this report, we develop a risk tool for estimating the 20-year risk of developing diabetes among middle-aged and elderly individuals who are free of diabetes at baseline. Our risk estimates enable an adjustment for the competing risk of non-diabetes death, and simultaneously include lifestyle behaviours, psychological factors, cognitive function and physical conditions simultaneously. The tool is based on the Beijing Longitudinal Study on Ageing, which has contributed to the development of a 10-year risk score algorithm for coronary artery disease using a sub-distribution hazards model^27^, and offers 20 years of rigorous surveillance data for diabetes occurrence.

## Results

### Baseline Characteristics and Follow-up

We followed 1857 participants who did not have diabetes at baseline for a median 10.9 (Interquartile range: 8.0–15.3)-years period. The average age at baseline was 69.00 ± 8.81 years for women and 69.88 ± 8.55 years for men at baseline. At the end of year 2012, there were 144 documented cases of incident diabetes, and 919 deaths from non-diabetes. Approximately 4.7% of the participants were lost to -follow-up (n = 87). The incidence density of diabetes was 7.908/1000 person-years. The cumulative incidence function (CIF) of incident diabetes was 11.60% after adjusting for the competing risks of non-diabetes deaths. There were differences between the incident diabetes and non-diabetes groups in the baseline distribution of age, disability, marital status, self-assessment of health status, blood lipids, and physical exercise (P < 0.05) (Table 1). The baseline characteristics of the subjects based on non-diabetes and diabetes events for men and women at the baseline are also provided in Table 1. The sensitivity analysis showed that there were no statistically significance differences in the distribution of baseline characteristics between those lost to follow-up and those retained.

### Diabetes Risk Prediction Model

Univariate analyses were used to regress the sub-distribution hazard of diabetes incidence on all twelve candidate variables, and the estimated regression coefficients, estimated regression sub-hazard ratios, estimated 95% confidence intervals, and the statistical significance of the estimated regression coefficients are reported in Table 2. After accounting for competing risk events in the risk set, standard diabetes risk factors (female gender, age, overweight/obesity, IFG, poor self-assessment of health, divorced or single, and high blood lipids) were significant in the univariate analysis (*P* < 0.05). Then, all significant variables in the univariate analyses were entered into the multivariate prediction model; five variables were retained after backward selection (Table 2). In the multivariate prediction model, after all adjustments, a greater risk of diabetes incidence was associated with impaired FPG (SHR = 1.99, 95% CI = 1.37–2.90), poor self-assessment of health (SHR = 1.73, 95% CI = 1.19–2.51), overweight (SHR = 2.15, 95% CI = 1.44–3.21) or obesity (SHR = 1.96, 95% CI = 1.27–3.03), and less physical activity (SHR = 1.39, 95% CI = 1.01–1.91). The bootstrap-adjusted regression coefficients, SHR and score of the sub-distribution hazards model are presented in Table 3.

### Calibration, Discrimination, Reclassification, and Internal Validation

**Figure 1:**
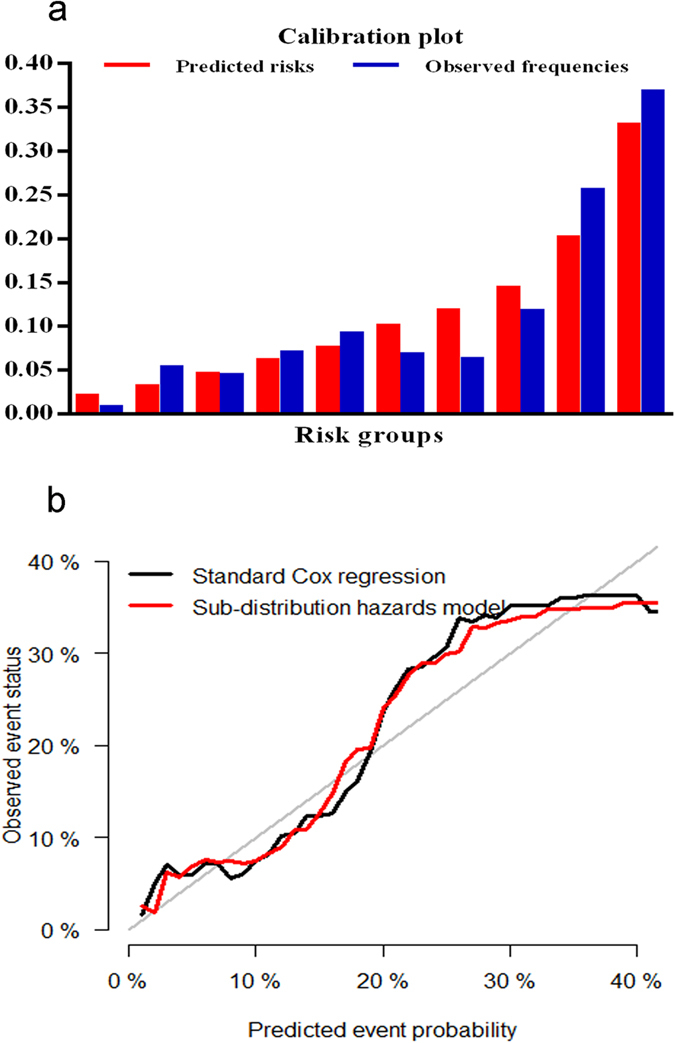
Calibration plots by deciles for diabetes prediction models of 20-year risk, adjusted for the competing risk of non-d1iabetes death: (**a**) the bar plot; (**b**) the line plot.

**Figure 2:**
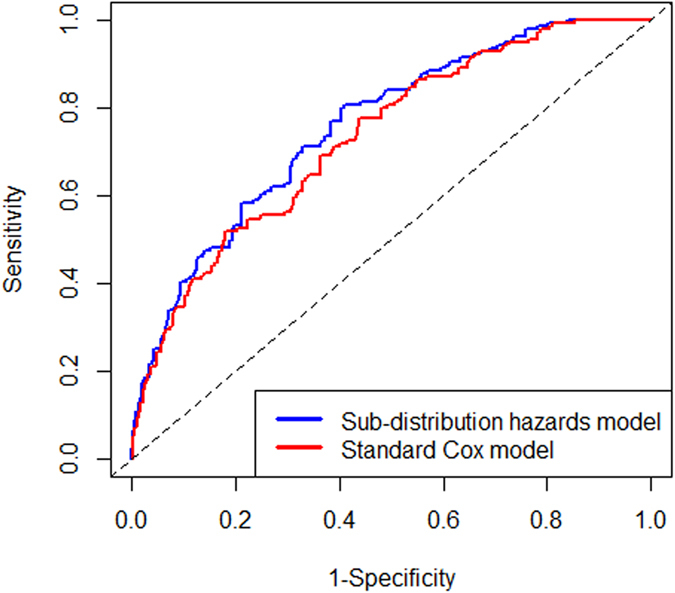
ROC curves for diabetes risk prediction model.

**Figure 3:**
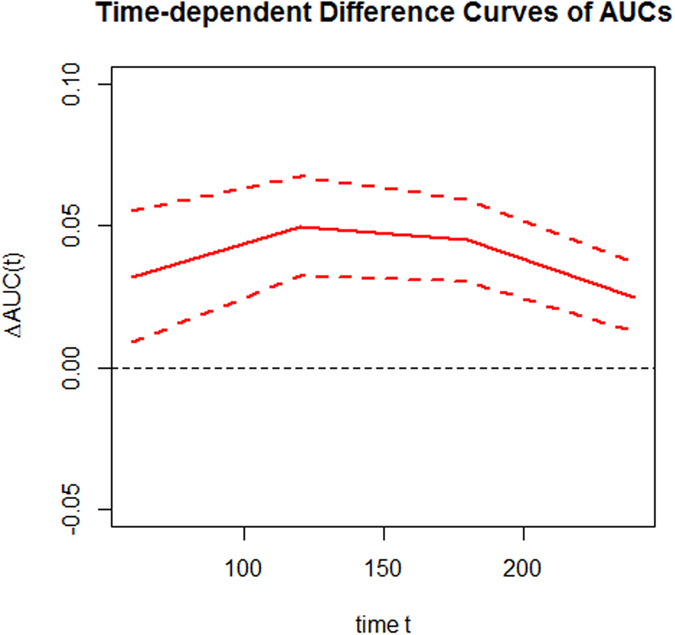
Differences curves of AUCs for 2 diabetes risk prediction models.

The calibration plot of the sub-distribution hazards model showed good calibration (Hosmer-Lemeshow test, chi-square = 4.544, *P* value = 0.805), and the actual diabetes risk in the BLSA cohort was similar to the predicted risk (Fig. 1). The sub-distribution hazards model performed better in terms of discrimination and calibration than Cox proportional hazards model. The area under the ROC curve (AUC) value were 0.76 (95% *CI*: 0.72–0.80) and 0.73 (95% *CI*: 0.69–0.77) for the sub-distribution hazards model and Cox proportional hazards model, respectively (Fig. 2). The AUC values of the sub-distribution hazards model were better than those of the Cox proportional hazard model at t = 20 years (Z = 4.30, *P* = 0.00002). The difference value of AUCs between sub-distribution and Cox proportional hazard models were more than zero (*P* = 0.307) (Fig. 3). After internal validation by bootstrapping, the optimism-corrected AUC of the sub-distribution hazards model at t = 20 years was 0.78 (95% CI: 0.69–0.87), and the optimism-corrected AUC of the Cox proportional hazard model at t = 20 years were 0.74 (95% CI: 0.65–0.84), suggesting a well-validated model.

### Additional value of self-rated health

**Figure 4:**
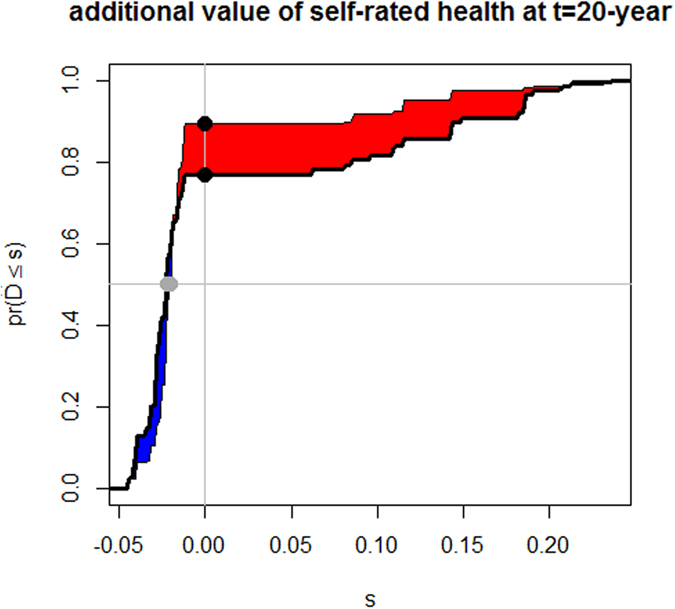
The additional value of self-assessment of health as assessed by the paired difference of risk scores at t = 20 years.

The additional variable self-rated health was assessed by the paired difference of risk scores. The empirical distribution function of the change in estimated risk scores for subjects who had events (thick solid line) and those who were event-free (thin solid line) was assessed (Fig. 4). The difference between the areas under the two curves is IDI, and the distances between the two black dots and between the two grey dots represent the continuous NRI and median improvement, respectively. The estimations of IDI and NRI were 0.019 (95% *CI*: 0.002–0.054; *P* = 0.024) and 0.124 (95% *CI*: 0.032–0.236; *P* = 0.028), respectively, at t = 20 years. The median increment in the risk score after including self-rated health status in the prediction model was −0.002 (95% CI: −0.008–0.005; *P* = 0.351) at t = 20 years.

### Diabetes Risk Score Tool

Finally, we developed a simple risk score tool to estimate the 20-year diabetes risk for each individual using the baseline cumulative incidence function and the bootstrap-adjusted regression coefficients of the sub-distribution hazards model (Table 4). The score ranges from −4 to 38, and is positively related to the predicted risk of developing diabetes by linear regression (*P*_for trend_ < 0.001). The competing-risk-based score exhibited a reasonable sensitivity of 0.74 and specificity of 0.65, with an optimal cut-off value of 19 marking the difference between low-risk and high-risk patients at t = 20 years.

## Discussion

Using a community-based sample with a 20-year follow-up, we have constructed a multivariable risk factor algorithm applying a competing risk model that can be used to predict an individual’s risk and provides a helpful guide to identifying the groups at high risk for diabetes among adults over 55 years of age. To the best of our knowledge, this is the first community-based diabetes prediction model considering competing risk to be developed for an elderly population in China.

In terms of discrimination and calibration, the competing risk model is superior to Cox proportional hazard model. The competing risk analysis and Cox proportional hazard model may show no relevant differences when the mortality rate is low. From a statistical perspective, these models are not comparable, as they model different endpoints (cumulative incidence versus cause specific hazard). The present study extends and expands on the previous general diabetes risk models by adding a new risk factor, and the prediction model including self-rated health status was superior to the model without it. A user-friendly risk score tool predicting the 20-year probability of diabetes was developed.

Currently, the Finnish Diabetes Risk Score (FINDRISK)^7^, Framingham DM risk score^8^, Cambridge Diabetes Risk Score^11^, and German Diabetes Risk score^13^ are the most widely used scores in clinical guidelines. In addition, there are a number of other important risk algorithms or functions^28^. However, a prediction model specifically designed for the risk of incident diabetes in the Chinese elderly population is not currently available, especially one considering the competing risk. Our risk prediction model provided a feasible tool for identifying the high-risk individuals among the elderly in Beijing.

To the best of our knowledge, this is the first community-based diabetes prediction model considering competing risk that has been developed for the elderly population in China. It should be emphasized is that the general model evaluation methods are not applicable for competing risk models, calibration plots, net reclassification index (NRI), and integrated discrimination improvement (IDI) were calculated, and these values were adjusted for the competing risk of non-diabetes death.

The AUCs of previous diabetes risk scores for elderly adults ranged from 0.71 to 0.78 in their original population^9^,^10^. Our score based on the competing risk model showed a moderately high AUC value of 0.76 (95% CI: 0.72–0.80). Of note, a model with an AUC value less than 0.80 for predicting incident diabetes may have limited clinical utility. However, all predictors included in our scores are readily available clinical variables. If further predictors related to blood test results were included, the scores would likely show an improved performance.

In our score based on the competing risk model, age is the strongest predictor of incident diabetes (a contribution of 15 points). Individuals aged 55 to 65 years have the highest risk of developing diabetes in our scores (accounting for 39.47% of the total score based on the competing risk model), followed by individuals aged ranging from 66 to 75 years. Similarly results were found in the Guangzhou Biobank Cohort Study (GBCS), which was a 4.1-year population-based follow-up of 16,043 Chinese aged 50 years or above^10^.

BMI is the second-strongest predictor in our scores, and has been included in most of the published scores used to predict incident diabetes^10^. In our scores, the FPG variable is the third-strongest predictor after BMI (a contribution of 7 points). This result is roughly consistent with previous reports^9^. The value representing impaired fasting glucose (IFG) has been defined to be from 6.1 to 6.9 mmol/L. It is unsurprising that individuals with IFG have a high risk of developing diabetes. The risk of incident diabetes increased with high FPG levels.

Physical activity is also an important predictors of incident diabetes, and environmental pathways may be able to account for this relationship^13^. It has been demonstrated that interventions that include increases in physical activity are able to reduce the incidence of diabetes in high risk adults^29^,^30^. Another reason for this finding is that participants who frequently exercise are more likely to be aware of their blood glucose levels than people who never or rarely exercise.

We are the first to include self-rated health status in a diabetes prediction score. The competing-risk-based score included the self-rated health status and was assigned 6 points. Self-rated health (SRH) is a reflection of social, psychological, and biologic dimensions; it is one of the most widely used yet poorly understood measures of health^31^. In the present study, SRH was based on individuals’ assessment of their health status compared with that of peers their age. Similar to our results, SRH scores provide additional valuable information for risk prediction in patients with diabetes^32^, and it has also been recommended as a tool for assessing cardiovascular disease risk assessment^33^. Thus, diabetes guidelines should extend their focus on clinical and social aspects of diabetes to include questions on patient’s SRH^34^.

There were some limitations to our study. First, we did not included waist circumference. However, the Guangzhou Biobank Cohort Study showed that using waist circumference or waist-to-hip ratio instead of BMI did not substantially improve the discrimination substantially^10^. Second, due to the long-term follow-up, follow-up biases could easily have been introduced. However, the sensitivity analysis showed that there were no statistically significant differences in the distribution of baseline characteristics between those lost to follow-up and those who remained in the study. In addition, because cases of diabetes were identified through reexamination and questionnaires, diabetes onset occurred prior to diagnosis.

## Conclusion

We constructed a multivariable risk score using a community-based sample with a 20-year follow-up that can be used to predict an individual’s risk for diabetes among adults over 55 years of age. To the best of our knowledge, this is the first community-based diabetes risk score to consider competing risk developed for an elderly population in China. Further studies are needed to test this score in other population samples of China.

## Methods

### The BLSA study

According to the 10% sampling data from Beijing in China’s fourth census, a three-stage stratification (i.e., natural living environment, education level and degree of ageing) random-clustering sampling procedure was conducted to ensure the representativeness of the elderly population in Beijing^35^. The communities included were located in Huairou district, Daxing district, and Xuanwu district, which are representative of the northern, middle and southern region of Beijing, respectively. Periodic health examinations were performed every 2–3 years (in year 1992, 1994, 1997, 2000, 2004, 2007, 2009 and 2012) and included questionnaire interviews, anthropometric measurements, clinical examinations, and laboratory assessments. We used complete data for the period from 1992 to 2012 in the study. A community-based cohort of 2101 people (1037 men and 1064 women, 55–96 years old) were recruited for the BLSA (Beijing Longitudinal Study on Ageing) from August 1992 to December 2012, and this study was managed by Xuanwu Hospital of Capital Medical University in Beijing, China. In total, 244 subjects were excluded because of they had either a baseline FPG level higher than 7.0 mmol/L (126 mg/dl) or a history of diabetes (as informed by a physician) or because they were taking antidiabetic medicine. This left 1857 participants (925 men and 932 women) who did not have diabetes at baseline for the analysis.

The study followed the guidelines of the Helsinki Declaration and was approved by the ethics committee of Xuanwu Hospital, Capital Medical University. Written informed consent was obtained from all participants.

### Assessment of risk factors and outcomes

The candidate baseline variables presented in Table 1 were chosen for their common availability and use in previous diabetes prediction models. The demographic characteristics and information on dietary habits, lifestyle, psychological factors and physical condition were obtained using questionnaires with a high degree of reliability and accuracy^36^; the questionnaires were administered by hospital research doctors of the hospitals who were specifically trained for the job. The questionnaires were designed by the Beijing Geriatric Clinical and Research Centre and the Australian Geriatric Research Centre of Flinders University. The measurement and classification of each category variable have been reported elsewhere in detail^35^.

A food frequency questionnaire was conducted for the dietary assessment^37^. Then, a latent class model was constructed and the best model was selected according to the value of the Bayesian information criterion. Based on the posterior probability (representing the frequency of food intake), dietary habits were divided into three latent groups: sufficient nutrition, intermediate-type and meat-based diet. Self-reported smoking, drinking, residence, and health status and the frequency of physical activity were evaluated by questionnaires with a high degree of reliability and accuracy. If the elderly exercised almost every day, this was defined as exercising frequently. The activities included Qi Gong, TaiChi, walking, running/jogging, dancing, etc.

Age was categorized into three sub-groups: 55 to 65 years, 66 to 75 years, and ≥76 years. Marital status was divided into two categories: married and unmarried. Height, weight, hip circumference, and waist circumference (2.5 cm above the umbilicus) were measured in the standing position without heavy clothing to the nearest 0.1 cm or 0.1 kg by nurses who were responsible for annual routine health examinations. BMI was calculated according to the equation BMI = weight (kg)/height (m)^2^ and was classified based on the common Chinese criteria^38^, i.e., thin corresponding to BMI < 18.5 kg/m^2^, normal to 18.5 ≤ BMI < 24.0 kg/m^2^, overweight to 24.0 ≤ BMI < 28.0 kg/m^2^, and obese to BMI ≥ 28.0 kg/m^2^.

Blood pressure (BP) was measured twice on the left arm of the seated participants with a mercury sphygmomanometer and an appropriately sized cuff; the average of the blood pressure measurements was constituted the examination blood pressure value. The two BP measurements were obtained with a 5-minute interval. If the two measurements differed by more than 5 mmHg, an additional reading was taken, and the final, average of the readings was used for the analysis. BP was classified into two groups: high (systolic blood pressure >140 mmHg or diastolic blood pressure >90 mmHg) and normal blood pressure.

Blood samples were collected after an overnight fast of at least 12 hours. FPG (Fasting plasma glucose), total cholesterol (TC), triglycerides (TG), high-density lipoprotein cholesterol (HDL-C), and low-density lipoprotein cholesterol (LDL-C) were subsequently determined with standardized enzymatic methods. Based on the standard of impaired FPG and dyslipidaemia, a FPG level of 6.1 to 6.9 mmol/L (109.8–125.9 mg/dl) was considered to impaired fasting glucose (IFG)^39^. A TC level of 5.18 mmol/L (200 mg/dL) or greater, a TG level of 1.7 mmol/L (150 mg/dL) or greater, a HDL-C level less than 1.03 mmol/L (40 mg/dL) in men and 1.29 mmol/L (50 mg/dL) in women, or an LDL-C level of more than 3.35 mmol/L (130 mg/dL) were considered to indicate dyslipidemia^40^.

The outcome of interest was the first incidence of diabetes at follow-up. This was identified according to either a self-reported history of diabetes diagnosis, or the use of antidiabetic medicine after the baseline examination, or a measured FPG level ≥ 7.0 mmol/L (126 mg/dl) at any of the periodic examinations. The date of diagnosis (incidence) was defined as the date of the examination visit when a new case of diabetes was identified or the diagnosis date on the most recently documented diabetes history collected by the questionnaire, whichever came first. Survival status was determined through interviews with surviving household members or neighbours when surviving household members were unavailable. The information was verified by a subset of participants based on household registration records. Cause of death was determined according to the International Classification of Disease (ICD), ninth revision (ICD-9 or ICD-10). Non-diabetes death, including from cardiovascular diseases, cancers and other causes, was classified as competing events.

### Statistical Analysis

Time of follow-up was calculated from the return date of the 1992 questionnaire until either incidence of diabetes, death, loss to follow-up, or the end of follow-up (December 2012), whichever came first. Considering the extensive length of the follow-up and the potential bias due to the competing risk of non-diabetes mortality, we employed a sub-distribution hazards model to adjust for the risk estimates of the competing risk of non-diabetes death as a competing risk^25^. The sub-distribution hazards model calculated the cumulative incidence of diabetes in the following manner:


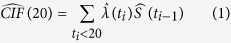


In equation (1), the quantities under summation denote the instantaneous hazard of diabetes at event time t_*i*_ and the survival rate from non-diabetes death past event time t_*i−1*_.

Sub-distribution hazards models were fitted to predict the risk of developing diabetes using package cmprsk and package crrstep in R software, which adjusted for clinical and biochemical variables. In the first step, univariate sub-distribution hazards models were used to regress the sub-distribution hazard of diabetes incidence on all nineteen candidate variables, and the variables with a statistical significance of the estimated regression coefficients of *P* > 0.20 were removed. Then, all significant variables were included to develop the multivariate prediction model with backward selection. In the third step, the remaining variables were included to build the final prediction model. For each model, sub-distribution hazard ratio (SHRs) and 95% confidence intervals (95% CIs) were calculated to estimate the relative risk.

Self-rated health is an important risk factor for diabetes, as confirmed in some studies^22^,^41^. However, no diabetes risk prediction models considered the impact of self-assessed of health status. Therefore, the diabetes risk prediction model in this study accounted for self-assessment of health status. We did not account for the interaction terms between the independent variables. All continuous variables included in the model were categorized, and thus the estimated contribution of these factors to diabetes risk could be expressed through simplified point scores assigned to each for the category. In addition, β-coefficients were calculated to determine points for each risk factor by multiplying the β-coefficients by 10 and rounding to the nearest integer. The sum of these points for each model was further calculated to predict the hazard of the incidence of diabetes over a mean follow-up period of 9.81 years for each person.

After the prediction models were developed, it was critical to evaluate their performance. The receiver operating characteristic (ROC) curve and areas under the ROC curves (AUCs, also referred to as C statistics) were used to evaluate the discriminative ability of the sub-distribution hazards models^42^ and were obtained by the ROCR package in R (R Foundation for Statistical Computing, Vienna, Austria)^43^. The cut-off point was estimated by calculating the value that minimizes the Euclidean distance between the ROC curve and the upper left corner of the graph. The calibration of the model was assessed graphically by comparing the predicted probability of the observed probability across the 10 deciles of predicted risk^44^, which was performed with the R package pec. Calibration refers to the agreement between observed outcomes and predictions. The more range there is between 10 deciles, the better discriminating the model. Hosmer-Lemeshow test was used to indicate the goodness of fit.

Additionally, internal validation was supported by estimating the potential of over- fitting and the optimism of the models^45^, which was performed by applying bootstrap resampling 1000 times with R package pROC. The bootstrap optimism-corrected AUC was computed by subtracting the optimism from the original AUC. Bootstrap-adjusted regression coefficients better reflect what can be expected when the model is tested or applied in new individuals from the same theoretical source population^45^. However, no internal validation methods can substitute for external validation.

Recently, some novel alternatives to the area under the receiver operating characteristic curve, such as net reclassification improvement (NRI) and integrated discrimination improvement (IDI), have been proposed^46^ to measure the improvement from the new risk factor in the prediction. The NRI and IDI are two new metrics used to the formally assess new risk factors, to supplement the improvement in the AUC, and were assessed using the R package of survIDINRI. All p-values reported were two-sided. Two-independent sample chi-square tests were in SAS software (Version 9.2, SAS Institute Inc., Cary, NC).

## Additional Information

**How to cite this article**: Liu, X. *et al.* A competing-risk-based score for predicting twenty-year risk of incident diabetes: the Beijing Longitudinal Study of Ageing study. *Sci. Rep.* **6**, 37248; doi: 10.1038/srep37248 (2016).

**Publisher’s note:** Springer Nature remains neutral with regard to jurisdictional claims in published maps and institutional affiliations.

## Figures and Tables

**Table 1:** Baseline characteristics between participants of incident diabetes and non-diabetes from the BLSA study.

Characteristic	Total (n = 1985)			Men (n = 925)			Women (n = 932)		
	Diabetes (%)	Non-diabetes (%)	*P*	Diabetes (%)	Non-diabetes (%)	*P*	Diabetes (%)	Non-diabetes (%)	*P*
sex			0.042	—	—	—	—	—	—
men	60 (6.49)	865 (93.51)							
women	84 (9.01)	848 (90.99)							
age-group			<0.001			<0.001			<0.001
55–65	93 (13.92)	575 (86.08)		40 (11.49)	308 (88.51)		53 (16.56)	267 (83.44)	
66–75	39 (5.76)	638 (94.24)		17 (4.90)	330 (95.10)		22 (6.67)	308 (93.33)	
>=76	12 (2.34)	500 (97.66)		3 (1.30)	227 (98.70)		9 (3.19)	273 (96.81)	
marital status			0.001			0.023			0.015
divorced or single	30 (4.88)	585 (95.12)		11 (3.77)	281 (96.23)		19 (5.88)	304 (94.12)	
married	114 (9.18)	1128 (90.82)		49 (7.74)	584 (92.26)		65 (10.67)	544 (89.33)	
self-rated of health			0.015			0.77			0.002
not health	36 (11.04)	290 (88.96)		12 (6.98)	160 (93.02)		24 (15.58)	130 (84.42)	
health	108 (7.05)	1423 (92.95)		48 (6.37)	705 (93.63)		60 (7.71)	718 (92.29)	
fasting plasma glucose		<0.001						0.002	
impaired	35 (13.62)	222 (86.38)		0	0		35 (13.62)	222 (86.38)	
normal	109 (6.81)	1491 (93.19)		60 (6.49)	865 (93.51)		49 (7.26)	626 (92.74)	
blood lipid			0.001			0.072			0.009
abnormal	49 (11.48)	378 (88.52)		18 (9.33)	175 (90.67)		31 (13.25)	203 (86.75)	
normal	95 (6.64)	1335 (93.36)		42 (5.74)	690 (94.26)		53 (7.59)	645 (92.41)	
blood pressure			0.854			0.990			0.869
abnormal	70 (7.87)	819 (92.13)		28 (6.50)	403 (93.50)		42 (9.17)	416 (90.83)	
normal	74 (7.64)	894 (92.36)		32 (6.48)	462 (93.52)		42 (8.86)	432 (91.14)	
education level			0.087			0.089			0.572
college or above	66 (6.75)	912 (93.25)		28 (5.29)	501(94.71)		38 (8.46)	411 (91.54)	
high school or below	78 (8.87)	801 (91.13)		32 (8.08)	364 (91.92)		46 (9.52)	437 (90.48)	
body mass index			0.187			0.988			0.133
thin	37 (10.16)	327 (89.84)		11 (6.92)	148 (93.08)		26 (12.68)	179 (87.32)	
normal	69 (6.68)	964 (93.32)		35 (6.31)	520 (93.69)		34 (7.11)	444 (92.89)	
overweight	20 (8.13)	226 (91.87)		7 (6.31)	104 (93.69)		13 (9.63)	122 (90.37)	
obesity	18 (8.41)	196 (91.59)		7 (7.00)	93 (93.00)		11 (9.65)	103 (90.35)	
area			0.322			0.139			0.631
mountain	29 (9.86)	265 (90.14)		20 (9.26)	196 (90.74)		9 (11.54)	69 (88.46)	
rural	36 (7.68)	433 (92.32)		18 (6.34)	266 (93.66)		18 (9.73)	167 (90.27)	
urban	79 (7.22)	1015 (92.78)		22 (5.18)	403 (94.82)		57 (8.52)	612 (91.48)	
diet			0.167						0.463
balanced	55 (8.97)	558 (91.03)		17 (6.88)	230 (93.12)	0.248	38 (10.38)	328 (89.62)	
middle status	63 (6.61)	890 (93.39)		26 (5.36)	459 (94.64)		37 (7.91)	431 (92.09)	
extra serving of meat	26 (8.93)	265 (91.07)		17 (8.81)	176 (91.19)		9 (9.18)	89 (90.82)	
physical activity			0.012			0.114			0.026
not frequently	73 (9.63)	685 (90.37)		33 (7.89)	385 (64.82)		40 (11.76)	300 (88.24)	
frequently	71 (6.46)	1028 (93.54)		27 (5.33)	480 (35.18)		44 (7.43)	548 (92.58)	

P values were based on two-independent sample chi-square test.

**Table 2:** Beta coefficients and HRs (95%CI) from sub-distribution hazards model based on the BLSA study.

Characteristic	Univariate analyses			Multivariate analyses (forward selection)		
	SHR (95 CI)	Coefficient	*P*	SHR (95 CI)	Coefficient	*P*
gender (men)						
women	1.50(1.08–2.08)	0.41	0.015	—	—	—
age-group, y						
≥76	Ref.	Ref.	—	Ref.	Ref.	
55–65	5.48 (3.01-10.00)	1.70	<0.001	4.37 (2.36-8.10)	1.48	<0.001
66–75	2.32 (1.21-4.43)	0.84	0.011	1.98 (1.02-3.83)	0.68	0.043
body mass index, (kg/m^2^)						
18.0-23.9	Ref.	Ref.	—	Ref.	Ref.	
<18.0	0.60 (0.33–1.06)	−0.52	0.077	0.64 (0.36-1.14)	−0.44	0.131
24.0–27.9	2.58 (1.73–3.85)	0.95	<0.001	2.15 (1.44–3.21)	0.76	<0.001
≥28.0	2.35 (1.52–3.63)	0.86	<0.001	1.96 (1.27–3.03)	0.68	0.002
fasting plasma glucose (normal)						
6.1–7.0	2.12 (1.46–3.08)	0.75	<0.001	1.99 (1.37–2.90)	0.69	<0.001
self-rated health (healthy)						
unhealthy	1.57 (1.09–2.29)	0.45	0.017	1.73 (1.19–2.51)	0.55	0.004
Physical activity (frequently)						
not frequently	1.36 (1.00–1.96)	0.31	0.059	1.39 (1.01–1.91)	0.33	0.047
marital status (married)						
divorced or single	1.82 (1.22–2.71)	0.60	0.003	—	—	—
blood-lipid (normal)						
high	1.75 (1.24–2.46)	0.559	0.001	—	—	—

**Table 3:** Bootstrap-adjusted beta coefficients and SHRs (95%CI) from sub-distribution hazards model and risk scores for predicting incident diabetes based on the BLSA study.

Characteristic	SHR (95 CI)	Coefficient	*P*	Score
Age-group, y				
≥76	Ref.	Ref.	—	0
55–65	4.37 (2.34–8.18)	1.48	<0.001	15
66–75	1.98 (0.99–3.95)	0.68	0.054	7
Body mass index, (kg/m^2^)				
18.0–23.9	Ref.	Ref.	—	0
<18.0	0.64 (0.36–1.15)	−0.44	0.134	−4
24.0–27.9	2.15 (1.40–3.28)	0.76	<0.001	8
≥28.0	1.96 (1.24–3.01)	0.68	0.004	7
Fasting plasma glucose				
normal	Ref.	Ref.	—	0
IFG	1.99 (1.32–3.01)	0.69	0.001	7
Self-rated health				
healthy	Ref.	Ref.	—	0
unhealthy	1.73 (1.18–2.54)	0.55	0.005	6
Physical activity				
frequently	Ref.	Ref.	—	0
not frequently	1.39 (0.99–1.95)	0.33	0.060	3

**Table 4:** The risk score tool for diabetes using sub-distribution hazards model based on the BLSA study.

Deciles of points	20-year risk estimate (%)	No. of participants (%)
−4~−1	0.25	108 (5.82)
0~3	0.48	158 (8.51)
3~7	0.88	202 (10.88)
7~10	1.74	236 (12.72)
10~13	2.81	188 (10.13)
13~15	3.82	162 (8.73)
15 ≤ 17	5.06	219 (11.80)
17~20	7.03	198 (10.67)
20~24	8.79	180 (9.70)
24~38	16.41	205 (11.05)
P for trend	<0.001	
